# Expression of Steroid Receptors in Ameloblasts during Amelogenesis in Rat Incisors

**DOI:** 10.3389/fphys.2016.00503

**Published:** 2016-11-02

**Authors:** Sophia Houari, Sophia Loiodice, Katia Jedeon, Ariane Berdal, Sylvie Babajko

**Affiliations:** ^1^Paris Laboratory of Molecular Oral Pathophysiology, Centre de Recherche des Cordeliers, Institut National de la Santé et de la Recherche Médicale UMRS 1138, Université Paris-Descartes, Université Pierre et Marie Curie-ParisParis, France; ^2^Université Paris-Diderot, Unité de Formation et de Recherche d'OdontologieParis, France; ^3^Centre de Référence des maladies rares de la face et de la cavité buccale MAFACE hôpital Rothschild, AP-HPParis, France

**Keywords:** amelogenesis, steroid receptors, steroid hormones, endocrine disrupting chemicals, enamel mineralization

## Abstract

Endocrine disrupting chemicals (EDCs) play a part in the modern burst of diseases and interfere with the steroid hormone axis. Bisphenol A (BPA), one of the most active and widely used EDCs, affects ameloblast functions, leading to an enamel hypomineralization pattern similar to that of Molar Incisor Hypomineralization (MIH). In order to explore the molecular pathways stimulated by BPA during amelogenesis, we thoroughly investigated the receptors known to directly or indirectly mediate the effects of BPA. The expression patterns of high affinity BPA receptors (ERRγ, GPR30), of ketosteroid receptors (ERs, AR, PGR, GR, MR), of the retinoid receptor RXRα, and PPARγ were established using RT-qPCR analysis of RNAs extracted from microdissected enamel organ of adult rats. Their expression was dependent on the stage of ameloblast differentiation, except that of ERβ and PPARγ which remained undetectable. An additional large scale microarray analysis revealed three main groups of receptors according to their level of expression in maturation-stage ameloblasts. The expression level of RXRα was the highest, similar to the vitamin D receptor (VDR), whereas the others were 13 to 612-fold lower, with AR and GR being intermediate. Immunofluorescent analysis of VDR, ERα and AR confirmed their presence mainly in maturation- stage ameloblasts. These data provide further evidence that ameloblasts express a specific combination of hormonal receptors depending on their developmental stage. This study represents the first step toward understanding dental endocrinology as well as some of the effects of EDCs on the pathophysiology of amelogenesis.

## Introduction

The environment has become increasingly contaminated by various pollutants which may have a role in the modern burst of diseases. Among environmental toxicants, endocrine-disrupting chemicals (EDCs) have been associated over these past 50 years with many existing or emerging diseases including hormone-dependent cancers, diabetes, obesity, and decreased fertility (De Coster and van Larebeke, 2012; Maqbool et al., 2016). This is supported by numerous epidemiological surveys (De Coster and van Larebeke, 2012; Grindler et al., 2015; Ehrlich et al., 2016) and experimental studies (Brieño-Enríquez et al., 2015; Chevalier et al., 2015; Robinson and Miller, 2015; Maqbool et al., 2016; Palanza et al., 2016; Ziv-Gal and Flaws, 2016 for recent reviews). Among the thousands of EDCs, bisphenol A (BPA) is one of the most active and ubiquitous due to its wide use by the plastic industry. The consequences of exposure to BPA have been studied in detail in the development and pathophysiology of multiple organs including gonads, brain, pancreas, liver, heart, and adipose tissue, acting on different effectors of the steroid axis (Chevalier et al., 2015; Robinson and Miller, 2015; Palanza et al., 2016; Seachrist et al., 2016; Ziv-Gal and Flaws, 2016 for recent reviews). BPA has also been shown to induce enamel hypomineralization in rats (Jedeon et al., 2013). Amelogenesis follows a well-known sequence of cell proliferation, differentiation, maturation, and death characterized by specific gene-expression patterns (Nanci, 2012). Ameloblasts sequentially secrete enamel matrix proteins (amelogenin, enamelin, ameloblastin) and proteases (KLK4 and MMP20). The proteases degrade the enamel matrix allowing subsequent mineral crystal growth under the correct pH and ionic conditions [aided by several solute carriers (SLCs) and ion-handling proteins]. BPA modulates the expression of at least one enamel key gene at each stage of amelogenesis, including enamelin, KLK4, and SLC26A4 (Jedeon et al., 2013, 2016a). The resulting rat enamel defects may be scored as those observed in human Molar Incisor Hypomineralization (MIH; Jedeon et al., 2013), a recently described enamel pathology (Weerheijm et al., 2001; Weerheijm and Merjare, 2003). The teeth of rats exposed to BPA and those of humans affected by MIH share similar structural and biochemical abnormalities. Thus, exposure of rats to BPA is a good experimental model of MIH (Jedeon et al., 2013). MIH mostly affects permanent first molars and incisors which are the first teeth to mineralize, from the third trimester of fetal life to four-5 years after birth (Weerheijm et al., 2001), corresponding to the window of the highest susceptibility to EDCs. This enamel disease presents a similar epidemiological evolution to EDC-related diseases. It was almost non-existent before the 80s', but now affects ~15–18% of 6 to 9-year-old children (Jälevik, 2010; Jedeon et al., 2015). It may therefore constitute a marker of exposure to pollutants that disrupt amelogenesis. The mechanism of action of BPA is still unclear but seem to modulate directly or indirectly the activity of multiple receptors (Acconcia et al., 2015). Among them, BPA has been shown to bind the estrogen receptors (ERα and ERβ) (Delfosse et al., 2012), GPR30 (or GPER) (Pupo et al., 2012) and ERRγ with a high affinity (Liu et al., 2012). It also directly or indirectly interferes with the activity of the androgen receptor (AR), the progesterone receptor (PGR), the glucocorticoid receptor (GR), and the PPARγ (Acconcia et al., 2015; Rehan et al., 2015). The mechanism of action of BPA in dental cells is even less evident as its putative receptors are poorly defined in dental tissues, except for ERα (Jedeon et al., 2014a).

The aim of this study was to systematically investigate the expression pattern of the putative BPA receptors and members of their family during amelogenesis in order to understand the effects of BPA on enamel as well as those of other EDCs acting through these receptors. These data may thus help to decipher the physiological endocrine-mediated regulations of amelogenesis and enamel pathologies resulting from endocrine disruption. To date, only the vitamin D pathway has been investigated in dental cells (Berdal et al., 1993; Descroix et al., 2010; Woo et al., 2015). Dental endocrinology needs to be explored in depth to understand the pathways of hormones effects on dental growth and enamel quality.

## Materials and methods

### Animals and biological samples

Two month-old Wistar rats were purchased from Janvier France Sarl (Le Genest Saint Isle, France) and bred in our animal house. All animals were fed *ad libitum*, and maintained in accordance with the guidelines for the care and use of laboratory animals from the French Ministry of Agriculture (A-75-06-12).

Three groups of three 30 day-old male and three other similar groups of female rats were constituted and used in this study. Rats were anesthetized by isoflurane inhalation, killed, and their mandibles immediately dissected. The incisors were extracted and soft dental tissues microdissected as previously described (Jedeon et al., 2013). Briefly, dental epithelial cells from the secretion stage and the maturation stage were separately dissected using the molar reference line for isolation, removing the underlying 2 mm-tissue corresponding to the transition stage (Smith and Nanci, 1989). The incisor wasn't opened during enamel organ dissection thus avoiding contamination by the mesenchyme. The anatomically distinguishable cervical loop was dissected from the apical end of the incisor. Microdissection quality was validated by RT-PCR using Enamelin primers for the secretion stage, and KLK4 or SLC26A4 for the maturation stage; Jedeon et al., 2016a). The absence of contamination by the mesenchyme and bone was verified using osteocalcin primers.

### RNA extraction and gene expression profiling

RNAs were extracted from microdissected cervical loop, and secretion- and maturation-stage cells of rat enamel organ using the RNeasy® Protect Mini Kit (Qiagen-France) according to the manufacturer's procedure. Spectrophotometry was used to assess the concentration and purity of RNA by measuring absorbance at 260 nm with a NanoDrop 1000ănd RNA Integrity Number (RIN) (threshold > 9.5) with an Agilent Bioanalyzer, respectively. Reverse transcription was carried out with 1 μg total RNA for 50 min at 42°C, using a random primer oligodT primer mix, according to the manufacturer's instructions (Superscript II®—Invitrogen). Real-time quantitative PCR was performed using the CFX96 device (Bio-Rad Laboratories, Hercules, CA, United States). SYBER green fluorescence corresponding to neosynthesized amplicons was quantified at the end of each of the 45 PCR cycles corresponding to a denaturation step of 2 s at 95°C followed by a polymerization step of 30 s at 60°C. Each PCR was independently repeated in triplicate and the results normalized against those for the three selected reference genes, RS15, GAPDH, and TBP1, for which the expression did not vary under our experimental conditions. Details of the primers and the corresponding amplicon sizes are presented in Table 1. The standard curve method was used to calculate the values corresponding to the relative amounts of test and reference RNAs. Mean ratios of test RNA/standard RNA were calculated for each sample. Similar data were obtained using the ΔΔCt method.

**Table 1:** **Primer sequences used for RT-qPCR analyses**.

**Gene**	**Amplicon size**	**Primer sequences (5′- 3′)**
GAPDH	400 bp	GACCCCTTCATTGACCTCAACTAC
		AAGTTGTCATGGATGACCTTGGCC
RS15	315 bp	GGCTTGTAGGTGATGGAGAA
		CTTCCGCAAGTTCACCTACC
TBP1	101 bp	CACGAACAACTGCGTTGATC
		TTTTCTTGCTGCTAGTCTGGAT
AR	105 bp	ACCTGACCTGGTTTTCAATGAGTATC
		GTTATCTGGAGCCATCCAAACTCTT
ERR*γ*	252 bp	GCCCATCCAATGATAACCAC
		GTCTTGACAGAGTGCGTGGA
ERRß	345 bp	TCGTATCTACTGGTGGCCGA
		TACGAGCTGCAAGATGGCTC
ERR*α*	230 bp	CTCTGGCTACCACTACGGTG
		GCTTGTACTTCTGTCGGCCA
ER*α*	112 bp	CCAGCTACAAACCAATGCACCATC
		GGTCTTTTCGTATCCCGCCTTTC
ERß	92 bp	CCATGATCCTCCTCAACTCCAGTATGT
		CGCGTTCAGTAGGTGTGTCAGCTT
GPR30	584 bp	GCAGCGTCTTCTTCCTCACC
		ACAGCCTGAGCTTGTCCTG
GR	294 bp	GTCATTACGGGGTGCTGACA
		GGGTGAGCTGTGGTAATGCT
MR	130 bp	GGCTACCACAGTCTCCCTGA
		ACGTTGACAATCTCCATGTAG
PGR	108 bp	CGCCCTACCTCAACTACCTG
		ATGCTTCATCCCCGCAGATT
RXR*α*	282 bp	GTGGATCTTTGGGGTGCAGCGT
		ACTCCAAACAGAGGTGCCA
VDR	175 bp	ACGTGCCCCGGATCTGTGGA
		CTGGCAGTGTCGCCGGTTGT

RNAs extracted from microdissected maturation-stage cells of male rat enamel organ were used for microarray experiments performed with Affymetrix RatGene1.0 ST chip probes at the Genom'IC platform of Cochin Institute (Paris, France) to measure the relative level of each (steroid) receptor.

### Immunofluorescence assays

Dental tissues were fixed by immersion in a 4% paraformaldehyde solution for 4 h. After washing in PBS, the samples were dehydrated in ethanol, rinsed in clearene (Leica-France) and paraffin-embedded (Paraplast plus, Sigma). Serial 8 μm sections were cut using a microtome (RM 2145, Leica, France). Sections were deparaffinized and rehydrated in decreasing concentrations of ethanol. Slices were microwaved for 20 min, and the tissues permeabilized with 0.5% Triton X-100 for 10 min. Sections were then washed in PBS and blocked with 10% normal goat serum in PBS for 1 h at room temperature. Slices were incubated overnight at 4°C with primary rabbit polyclonal anti-AR (N-20:sc-816, Santa Cruz) (1:200), anti-VDR (ab3508, Abcam) (1:500), or anti-ERα (sc-542, Santa Cruz) (1:50) antibodies. Sections were incubated with secondary goat anti-IgG coupled to Alexa Fluor 594 antibody (A-11072, Life Technologies) (1:500) at room temperature for one h in the dark. After rinsing with PBS, sections were immersed in DAPI (010M4003-Sigma) (1:100000) for 5 min and finally mounted with Fluoromount (Southern Biotech, Clinisciences).

### Statistical analysis

RT-qPCR data resulting from three independent analyses of three RNA samples of each tissue (loop, secretion, maturation, mesenchyme, and other tissues used as references) are presented as means ± *SD*. and were analyzed with GraphPad Prism Software Version 5.0 (GraphPad Software Inc., La Jolla, CA) using One way Analysis of Variance followed by Bonferroni's correction. Compared values were considered to be significantly different when ^*^p <0.05, ^**^p <0.01, or ^***^p <0.001.

## Results

### Expression patterns of BPA putative receptors during amelogenesis

**Figure 1:**
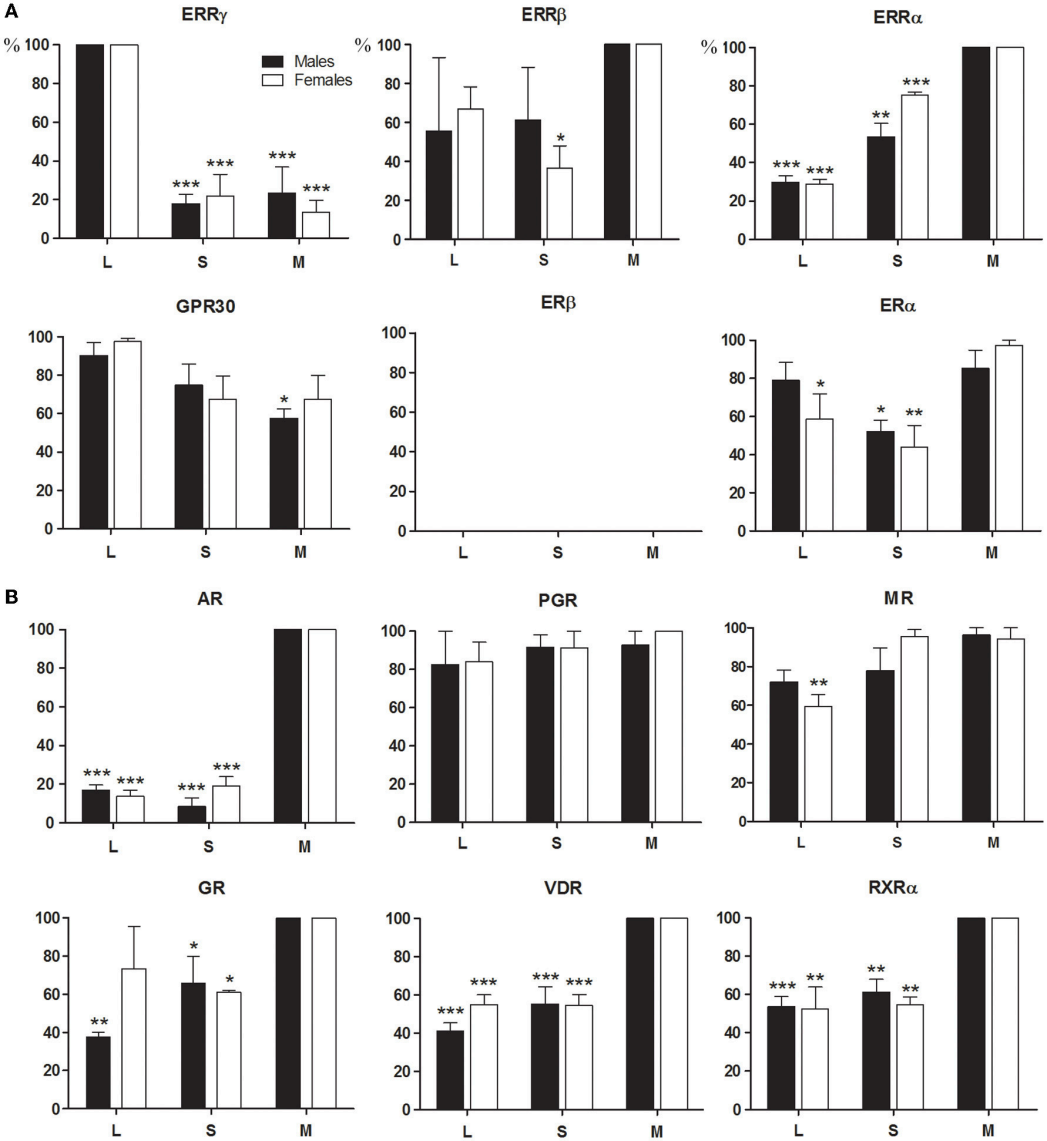
**Expression profiles of steroid receptors during amelogenesis**. RNAs extracted from microdissected rat enamel organ were analyzed by RT-qPCR after verifying the absence of mesenchymal and bone contamination. Dental cells from the secretion stage (S) and the maturation stage (M) were separately dissected using the molar reference line for isolation (See Materials and Methods). The cervical loop (L) that contains dental precursor cells, was anatomically distinguishable. The highest expression level ratio calculated for each studied and reference gene, using the standard curve method was set to 100% to compare data from the three independent experiments. Males (black bars) and females (white bars) were treated separately. The compared values were considered to be significantly different when ^*^p <0.05, ^**^p <0.01, ^***^p <0.001. **(A)** BPA receptors, ERRγ, and to a lesser extent GPR30 and ERα, were mainly expressed in the cervical loop, whereas ERRα and ERRβ were mostly expressed in the maturation stage. ERα and ERRβ expression pattern varied considerably between samples. ERβ was undetectable. **(B)** The other receptors able to mediate the effects of BPA were also expressed in the rat enamel organ, especially during the maturation stage. VDR and RXRα, two key receptors in amelogenesis, were also mostly expressed during the maturation stage.

We determined the specific pattern of expression for each high-affinity BPA receptor ERRγ, GPR30, ERα, and ERβ, and the other members of the ERR family, ERRα and ERRβ during amelogenesis by qPCR analysis of the enamel organ RNAs (Figure 1A).

Rat enamel organ cells expressed all the tested receptors except the ERβ, which was undetectable at all stages of amelogenesis (Figure 1A). The BPA receptors ERRγ, and to a lesser extent GPR30, were primarily expressed in early-stage ameloblasts (secretory and pre-ameloblasts). ERRγ expression was 5.0 to 6.7-fold higher in the cervical loop containing the precursors than in secretion and maturation stages containing differentiated ameloblasts. The other two members of the ERR family, the ERRα and ERRβ, were expressed throughout amelogenesis with a 3.6- and 1.3-fold accumulation in the maturation stage ameloblasts, respectively. The ERα presented a variable profile depending on the animal. Some animals expressed the ERα essentially in the cervical loop, whereas it was mostly in the maturation-stage ameloblasts in others.

Both males and females expressed similar levels of all receptors measured.

### Expression pattern of additional steroid receptors, GR, AR, MR, PGR, VDR, and retinoid receptors during amelogenesis

**Figure 2:**
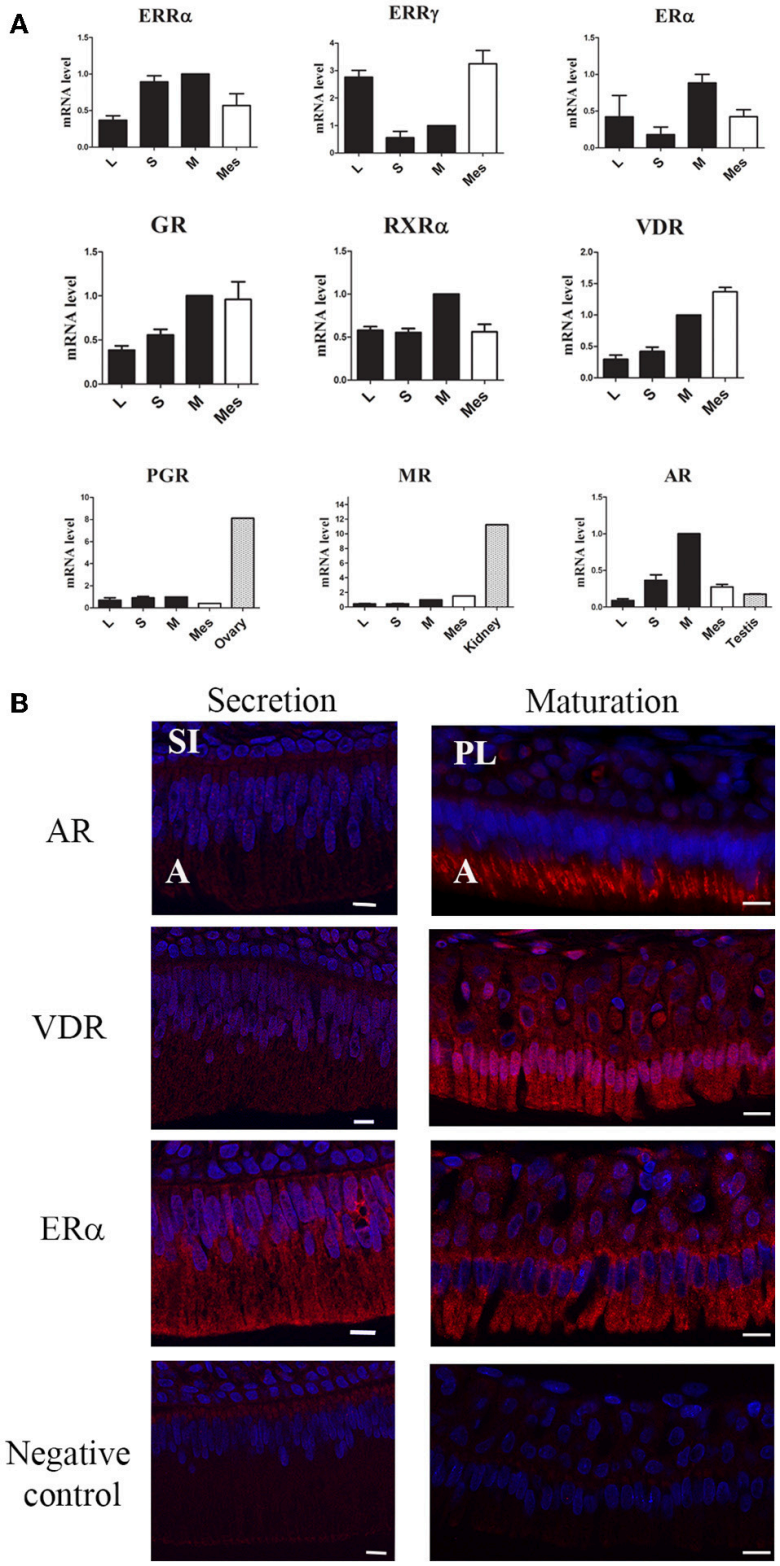
**Specificity of steroid hormone and VD receptor expression in maturation-stage ameloblasts. (A)** Expression levels calculated by the ΔΔCt method were compared between the cervical loop (L), secretion-stage cells (S), maturation-stage cells (M), mesenchymal cells (Mes) and other tissues used as references: testis for AR, kidney for MR, and ovary for PGR. The AR showed the most preferential expression in maturation-stage enamel tissue relative to all the other receptors tested with a level of expression even higher than that found in testis, used as the androgen responsive tissue. Results are from three independent analyses of three RNA samples of each tissue and are presented as the means ± *SD*. **(B)** Immunofluorescent assays for the AR, ERα, and VDR, three receptors involved in amelogenesis. The ER signal was very low in all cells of the enamel organ. The signals corresponding to the AR and VDR were clearly localized in maturation-stage ameloblasts (involved in enamel terminal mineralization). The AR and VDR were also slightly detected in the secretion-stage. A, ameloblasts; PL, papillary layer; SI, stratum intermedium. Scale bars, 10 μm.

We also measured the expression of all receptors known to be involved in the action of BPA, including the AR, PGR and GR/MR (Figure 1B). The AR exhibited the highest difference of expression which was 7.3-fold higher in maturation-stage than in early-stage ameloblasts. AR mRNA was mostly detected in maturation-stage epithelium where its level of expression was 3.6- and 5.7-fold higher than in the mesenchyme and in testis, respectively (Figure 2A). Immunofluorescence assays also showed the presence of the AR protein in dental epithelium, exclusively in maturation-stage ameloblasts, but not in secretion-stage ameloblasts, nor in cells of the papillary layer (Figure 2B). Among the different receptors investigated, its localization was the most specific, restricted to maturation-stage ameloblasts.

The other receptors were mostly expressed in maturation-stage ameloblasts with a 2.6-fold higher level of the GR than in the cervical loop (Figure 1B). The MR and PGR were also mostly expressed during the maturation stage, but with only small differences relative to other stages. The level of MR expression in the maturation-stage cells was 11.2-fold lower than in the kidney, and the level of PGR 8.1-fold lower than in the ovary used as positive controls (Figure 2A).

We also examined the expression patterns of the VDR and its partner the RXRα. Both VDR and RXRα mRNAs accumulated in the maturation-stage ameloblasts with a mean two-fold higher level than in the other compartments of enamel organ (Figure 1B). Immunohistological assays, showing the localization of the corresponding proteins, confirmed the RT-qPCR data with a signal for the VDR throughout the enamel organ, but stronger in mature ameloblasts (Figure 2B).

We observed no major differences between males and females (Figure 1B).

### Comparison of relative expression levels of steroid, BPA, retinoid, and vitamin D receptors in maturation stage ameloblasts

**Figure 3:**
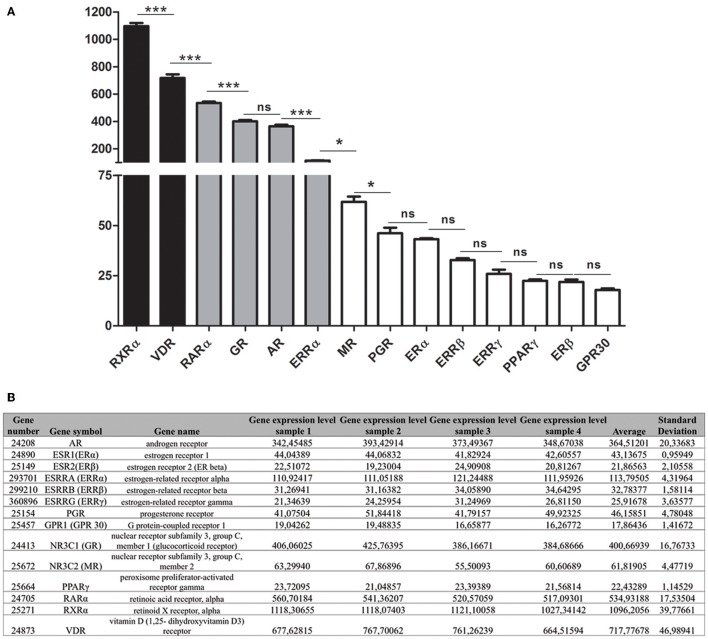
**Relative level of expression of steroid hormone and vitamin receptors during the maturation stage. (A)** The relative level of expression of each mRNA was determined by microarray analysis of RNAs extracted from maturation-stage enamel organ. Three main groups of receptors were distinguished: The RXRα and VDR were the most highly expressed (black bars); the GPR30, MR, PGR, ERα, ERRβ, and ERRγ weakly (white bars); and the RARα, GR, AR, and ERRα expression levels were intermediate (gray bars). PPARγ, GPR30 and ERβ were at the limit of the detection. Data resulted from microarray analyses of four RNA samples were presented as means ± *SD* and were compared using One way Analysis of Variance followed by Bonferroni's correction. The compared values were considered to be significantly different when ^*^p <0.05, ^***^p <0.001. ns, non significant. **(B)** Raw microarray data and statistical analysis for the calculated mean levels of expression of the studied receptors.

We determined the relative expression levels of the studied receptors in maturation-stage ameloblasts by microarray analysis. The most highly expressed receptors were RXRα, RARα, and VDR (Figures 3A,B). Maturation-stage ameloblasts also expressed all members of the ketosteroid receptors, GR, MR, AR, and PGR. GR and AR levels of expression were similar whereas MR and PGR were 5.9- and 7.9-fold lower, respectively.

The other receptors (ERα, ERRβ, ERRγ) were weakly expressed in maturation-stage ameloblasts: ERRγ was one of the least expressed, with mRNA level that was 27.7-fold less than the VDR (Figure 3). The ERβ, GPR30, and PPARγ, three other putative BPA receptors were almost undetectable.

## Discussion

The effects of vitamin D (VD) on bone and enamel mineralization are well-known, but little is known about all other endocrine regulations of dental growth and mineralization. Recent reports showing the effects of EDCs on enamel mineralization (Bloch-Zupan et al., 1994; Alaluusua et al., 2004; Jan et al., 2007; Jedeon et al., 2014b) suggest that amelogenesis may be regulated by endogenous steroid hormones. The present study shows that many steroid receptors are expressed by ameloblasts with a specific pattern depending on cell proliferation and differentiation, making ameloblasts responsive cells to steroid hormones. The VDR, which binds VD and forms active heterodimers with the RXRα, was the most highly expressed nuclear receptor along with the RXRα throughout amelogenesis. This is in accordance with previous data showing the presence of VDR (Berdal et al., 1993) and RARα/RXRα (Bloch-Zupan et al., 1994) in enamel organ cells, reflecting the importance of VD and vitamin A/retinol in tooth development reported many years ago. VDR/RXR heterodimers control ameloblast differentiation and the expression of key enamel genes such as amelogenin and calbindin D 28k (Berdal et al., 1993; Papagerakis et al., 1999). They were also the most highly expressed nuclear receptors in mesenchymal cells, including odontoblasts, in accordance with previously published data showing the effects of VD on dentin (Davideau et al., 1996). We also detected the GR, AR, and ERRα, among the most highly expressed steroid hormone receptors, throughout amelogenesis with the highest level of expression in maturation-stage ameloblasts. The role of ERRα, and more generally of ERRs, in amelogenesis is unknown. Corticoids affect enamel hardness and mineralization (Pawlicki et al., 1992), and a responsive element for GR (GRE) has been found in the amelogenin promoter (Gibson et al., 1997). Concerning the AR, it has already been detected in dental pulp cells (Dale et al., 2002; Inaba et al., 2013). In addition, our past work, as well as the present study, show that AR expression in the maturation-stage ameloblasts which is higher than in the secretion-stage and mesenchymal cells, suggesting a selective role of androgens in enamel final mineralization (Jedeon et al., 2016b). Testosterone is able to modulate the expression of enamel key genes present in maturation-stage ameloblasts such as SLC26A4 (or pendrin) and KLK4 (Jedeon et al., 2016b). Moreover, the higher level of AR expression in dental epithelium than in testis suggests that ameloblasts are responsive to plasmatic testosterone and thus androgen regulation of final enamel mineralization. This is likely not the case for the PGR and MR which levels of expression in enamel organ were 10 to 20-fold lower than in ovary and kidney, respectively.

The generally higher expression of steroid hormone receptors in the maturation-stage ameloblasts suggests a hormonal control of final enamel mineralization, and thus of enamel quality rather than enamel quantity. This has been experimentally demonstrated in rodent models for the VD/VDR. The deletion of the VDR leads to enamel hypomineralization even in the presence of normal levels of calcium and phosphate (Descroix et al., 2010). Indeed, low serum levels of VD during infancy is associated to caries (Schroth et al., 2014). Dental decay is a complex process involving many factors such as saliva, oral microbiota, and lifestyle, but enamel quality is also an important parameter. Elevated VD serum levels are negatively correlated to MIH (Kühnisch et al., 2015) and to EDC contamination (Johns et al., 2016), suggesting that MIH may be due, at least in part, to endocrine disruption. Epidemiological data have shown that contamination by PCBs and dioxin, two different classes of EDCs, may be associated with enamel hypomineralization (Alaluusua et al., 2004; Jan et al., 2007). Our previous experimental data showed that rats exposed to low-dose genistein and vinclozolin, as well as BPA, present enamel hypomineralization similar to human MIH (Jedeon et al., 2013, 2014b), which is both a hypomineralizing and hypoplasic enamel pathology (Jedeon et al., 2013). The selective affection of MIH suggests disruption during a narrow time window compatible with the steroid hormone secretion pattern during enamel mineralization. The clinical characteristics of enamel defects in MIH also suggest that BPA disrupts amelogenesis throughout the process. It may directly or indirectly modulate receptor activities, not only in maturation-stage ameloblasts, but also in pre-secretory and proliferating cells of the cervical loop. The ERα has already been shown to mediate, at least in part, the short-term mitogenic effects of BPA in pre-ameloblastic cells, but not genomic effects (Jedeon et al., 2014a). Similar non-genomic effects of BPA involving GPR30 activation has been shown in prostate cancer cells (Prins et al., 2014). The three high affinity BPA receptors, ERRγ, GPR30, and ERs, were very weakly expressed in the maturation-stage ameloblasts. They are mainly detected in proliferating epithelial and mesenchymal precursor cells of the loop, especially the ERRγ, which is the highest affinity receptor for BPA (Okada et al., 2008; Acconcia et al., 2015). The ERRγ is the *in vivo* receptor of BPA involved in the mineralization process of otoliths in zebrafish (Tohmé et al., 2014).

Despite the preferential impact of BPA in males, we detected no major differences between males and females in the hormone receptor expression patterns, or their expression levels. One possible explanation is that this sexual discrepancy may be due to disrupted levels of estrogens or androgens (Scinicariello and Buser, 2016). BPA exerts its anti-androgenic effects by preventing AR activation and lowering the levels of endogenous testosterone. BPA and other anti-androgenic EDCs may exert their anti-androgenic effects on final enamel mineralization through the AR expressed in maturation-stage ameloblasts (Jedeon et al., 2016b). Thus, the high testosterone levels in males following the birth, concomitant with amelogenesis, may cause a sexual dimorphism in enamel quality.

In conclusion, our data show that dental cells express many steroid receptors, of which the expression pattern depends on their stage of differentiation. This study provides clues for further studies of dental endocrinology which needs to be developed in depth to understand the effects of steroid hormone receptors and EDCs acting through such receptors on dental growth and enamel quality.

## Author contributions

SH, SL, and KJ: Contribution to the acquisition, analysis and interpretation of data, interpretation of data for the work, drafting the work, final approval of the version to be published, and agreement to be accountable for all aspects of the work in ensuring that questions related to the accuracy or integrity of any part of the work are appropriately investigated and resolved. AB and SB: Substantial contributions to the conception and design of the work, interpretation of data for the work, drafting the work, writing the paper, final approval of the version to be published, and agreement to be accountable for all aspects of the work in ensuring that questions related to the accuracy or integrity of any part of the work are appropriately investigated and resolved.

## Funding

This work was funded by the University Paris-Diderot, the French National Institute of Health and Medical Research (INSERM), the Institut Benjamin Delessert, and the Institut Français pour la Recherche Odontologique (IFRO).

### Conflict of interest statement

The authors declare that the research was conducted in the absence of any commercial or financial relationships that could be construed as a potential conflict of interest.

## References

[B1] Acconcia F. Pallottini V. Marino M. (2015). Molecular mechanisms of action of BPA. Dose Response 13:1559325815610582. 10.1177/1559325815610582 26740804 PMC4679188

[B2] Alaluusua S. Calderara P. Gerthoux P. M. Lukinmaa P. L. Kovero O. Needham L.. (2004). Developmental dental aberrations after the dioxin accident in Seveso. Environ. Health Perspect. 112, 1313–1318. 10.1289/ehp.6920 15345345 PMC1247522

[B3] Berdal A. Hotton D. Pike J. W. Mathieu H. Dupret J. M. (1993). Cell- and stage-specific expression of vitamin D receptor and calbindin genes in rat incisor: regulation by 1,25-dihydroxyvitamin D3. Dev. Biol. 155, 172–179. 10.1006/dbio.1993.1016 8380146

[B4] Bloch-Zupan A. Mark M. P. Weber B. Ruch J. V. (1994). *In vitro* effects of retinoic acid on mouse incisor development. Arch. Oral Biol. 39, 891–900. 10.1016/0003-9969(94)90021-3 7741659

[B5] Brie-o-Enríquez M. A. García-López J. Cárdenas D. B. Guibert S. Cleroux E. Děd L.. (2015). Exposure to endocrine disruptor induces transgenerational epigenetic deregulation of microRNAs in primordial germ cells. PLoS ONE 10:e0124296. 10.1371/journal.pone.0124296 25897752 PMC4405367

[B6] Chevalier N. Fénichel P. Bisphenol A. (2015). Targeting metabolic tissues. Rev. Endocr. Metab. Disord. 16, 299–309. 10.1007/s11154-016-9333-8 26820262

[B7] Dale J. B. Sarich S. L. Bretz T. M. Hatton J. F. Zachow R. J. (2002). Hormonal regulation of androgen receptor messenger ribonucleic acid expression in human tooth pulp. J. Dent. Res. 81, 360–365. 10.1177/154405910208100514 12097452

[B8] Davideau J. L. Papagerakis P. Hotton D. Lezot F. Berdal A. (1996). *In situ* investigation of vitamin D receptor, alkaline phosphatase, and osteocalcin gene expression in oro-facial mineralized tissues. Endocrinology 137, 3577–3585. 10.1210/endo.137.8.8754789 8754789

[B9] De Coster S. van Larebeke N. (2012). Endocrine-disrupting chemicals: associated disorders and mechanisms of action. J. Environ. Public Health 2012:713696. 10.1155/2012/713696 22991565 PMC3443608

[B10] Delfosse V. Grimaldi M. Pons J. L. Boulahtouf A. le Maire A. Cavailles V.. (2012). Structural and mechanistic insights into bisphenols action provide guidelines for risk assessment and discovery of bisphenol A substitutes. Proc. Natl. Acad. Sci. U.S.A. 109, 14930–14935. 10.1073/pnas.1203574109 22927406 PMC3443136

[B11] Descroix V. Kato S. Lézot F. Berdal A. (2010). Physiopathology of dental rickets in vitamin D receptor-ablated mice. J. Dent. Res. 89, 1427–1432. 10.1177/0022034510379603 20929724

[B12] Ehrlich S. Lambers D. Baccarelli A. Khoury J. Macaluso M. Ho S. M. (2016). Endocrine disruptors: a potential risk factor for gestational diabetes mellitus. Am. J. Perinatol. 33, 1313–1318. 10.1055/s-0036-1586500 27490770

[B13] Gibson C. W. Collier P. M. Yuan Z. A. Chen E. Adeleke-Stainback P. Lim J.. (1997). Regulation of amelogenin gene expression. Ciba Found Symp. 205, 187–197. discussion: 197–199. 9189625 10.1002/9780470515303.ch13

[B14] Grindler N. M. Allsworth J. E. Macones G. A. Kannan K. Roehl K. A. Cooper A. R. (2015). Persistent organic pollutants and early menopause in U.S. women. PLoS ONE 10:e0116057. 10.1371/journal.pone.0116057 25629726 PMC4309567

[B15] Inaba T. Kobayashi T. Tsutsui T. W. Ogawa M. Uchida M. Tsutsui T. (2013). Expression status of mRNA for sex hormone receptors in human dental pulp cells and the response to sex hormones in the cells. Arch. Oral Biol. 58, 943–950. 10.1016/j.archoralbio.2013.02.001 23490353

[B16] Jälevik B. (2010). Prevalence and diagnosis of Molar-Incisor- Hypomineralisation (MIH): a systematic review. Eur. Arch. Paediatr. Dent. 11, 59–64. 10.1007/BF03262714 20403299

[B17] Jan J. Sovcikova E. Kocan A. Wsolova L. Trnovec T. (2007). Developmental dental defects in children exposed to PCBs in eastern Slovakia. Chemosphere 67, S350–S354. 10.1016/j.chemosphere.2006.05.148 17250867

[B18] Jedeon K. Berdal A. Babajko S. (2015). The tooth, target organ of Bisphenol A, could be used as a biomarker of exposure to this agent, in Sources, Risks of Environmental Exposure and Human Health Effects, eds YG Bisphenol A. (New York, NY: Nova Science Publishers), 205–225.

[B19] Jedeon K. De la Dure-Molla M. Brookes S. J. Loiodice S. Marciano C. Kirkham J. (2013). Enamel defects reflect perinatal exposure to bisphenol A. Am. J. Pathol. 83, 108–118. 10.1016/j.ajpath.2013.04.004 PMC3703547 23764278

[B20] Jedeon K. Houari S. Loiodice S. Thuy T. T. Le Normand M. Berdal A.. (2016a). Chronic exposure to bisphenol A exacerbates dental fluorosis in growing rats. J. Bone Miner. Res. [Epub ahead of print]. 10.1002/jbmr.2879 27257137

[B21] Jedeon K. Loiodice S. Le Normand M. Houari S. Chaloyard J. Salhi K.. (2016b). Androgen receptor involvement in rat amelogenesis: an additional way for endocrine disrupting chemicals to affect enamel synthesis. Endocrinology. [Epub ahead of print]. 10.1210/en.2016-1342 27684650

[B22] Jedeon K. Loiodice S. Marciano C. Vinel A. Canivenc Lavier M. C. Berdal A.. (2014a). Estrogen and bisphenol A affect male rat enamel formation and promote ameloblast proliferation. Endocrinology 155, 3365–3375. 10.1210/en.2013-2161 25004094

[B23] Jedeon K. Marciano C. Loiodice S. Boudalia S. Canivenc Lavier M.-C. Berdal A.. (2014b). Enamel hypomineralization due to endocrine disruptors. Connective Tissue Res. 55, 1–5. 10.3109/03008207.2014.923857 25158179

[B24] Johns L. E. Ferguson K. K. Meeker J. D. (2016). Relationships between urinary phthalate metabolite and bisphenol A concentrations and vitamin D levels in U.S. Adults: National Health and Nutrition Examination Survey (NHANES), 2005-2010. J. Clin. Endocrinol. Metab. jc20162134. [Epub ahead of print]. 10.1210/jc.2016-2134 27648964 PMC5095248

[B25] Kühnisch J. Thiering E. Kratzsch J. Heinrich-Weltzien R. Hickel R. Heinrich J. (2015). GINIplus study group; LISAplus study group. Elevated serum 25(OH)-vitamin D levels are negatively correlated with molar-incisor hypomineralization. J. Dent. Res. 94, 381–387. 10.1177/0022034514561657 25503610 PMC4438736

[B26] Liu X. Matsushima A. Nakamura M. Costa T. Nose T. Shimohigashi Y. (2012). Fine spatial assembly for construction of the phenol pocket to capture bisphenol A in the human nuclear receptor estrogen related receptor γ. J. Biochem. 151, 403–415. 10.1093/jb/mvs008 22298789

[B27] Maqbool F. Mostafalou S. Bahadar H. Abdollahi M. (2016). Review of endocrine disorders associated with environmental toxicants and possible involved mechanisms. Life Sci. 145, 265–273. 10.1016/j.lfs.2015.10.022 26497928

[B28] Nanci A. (2012). Enamel: Composition, Formation, and Structure, in Ten Cate's. Oral Histology Development, Structure, and Function, 8th Edn, (Saint Louis: Elsevier Mosby), 122–164.

[B29] Okada H. Tokunaga T. Liu X. Takayanagi S. Matsushima A. Shimohigashi Y. (2008). Direct evidence revealing structural elements essential for the high binding ability of bisphenol A to human estrogen-related receptor-gamma. Environ. Health Perspect. 116, 32–38. 10.1289/ehp.10587 18197296 PMC2199305

[B30] Palanza P. Nagel S. C. Parmigiani S. Vom Saal F. S. (2016). Perinatal exposure to endocrine disruptors: sex, timing and behavioral endpoints. Curr. Opin. Behav. Sci. 7, 69–75. 10.1016/j.cobeha.2015.11.017 27019862 PMC4805122

[B31] Papagerakis P. Hotton D. Lezot F. Brookes S. Bonass W. Robinson C.. (1999). Evidence for regulation of amelogenin gene expression by 1,25-dihydroxyvitamin D(3) *in vivo*. J. Cell. Biochem. 76, 194–205. 10.1002/(SICI)1097-4644(20000201)76:2<194::AID-JCB4>3.0.CO;2-U 10618637

[B32] Pawlicki R. Knychalska-Karwin Z. Stankiewicz D. Jakób-Dolezal K. Karwan T. (1992). Disturbances of mineral metabolism in teeth of rats receiving corticosteroids for 3 generations. Folia Histochem. Cytobiol. 30, 75–78. 1483537

[B33] Prins G. S. Hu W. Y. Shi G. B. Hu D. P. Majumdar S. Li G.. (2014). Bisphenol A promotes human prostate stem progenitor cell self-renewal and increases *in vivo* carcinogenesis in human prostate epithelium. Endocrinology 155, 805–817. 10.1210/en.2013-1955 24424067 PMC3929731

[B34] Pupo M. Pisano A. Lappano R. Santolla M. F. De Francesco E. M. Abonante S.. (2012). Bisphenol A induces gene expression changes and proliferative effects through GPER in breast cancer cells and cancer-associated fibroblasts. Environ. Health Perspect. 120, 1177–1182. 10.1289/ehp.1104526 22552965 PMC3440081

[B35] Rehan M. Ahmad E. Sheikh I. A. Abuzenadah A. M. Damanhouri G. A. Bajouh O. S.. (2015). Androgen and progesterone receptors are targets for bisphenol A (BPA), 4-Methyl-2,4-bis-(P-Hydroxyphenyl)Pent-1-Ene–a potent metabolite of BPA, and 4-Tert-Octylphenol: a computational insight. PLoS ONE 10:e0138438. 10.1371/journal.pone.0138438 26379041 PMC4574962

[B36] Robinson L. Miller R. (2015). The impact of bisphenol A and phthalates on allergy, asthma, and immune function: a review of latest findings. Curr. Environ. Health Rep. 2, 379–387. 10.1007/s40572-015-0066-8 26337065 PMC4626318

[B37] Schroth R. J. Lavelle C. Tate R. Bruce S. Billings R. J. Moffatt M. E. (2014). Prenatal vitamin D and dental caries in infants. Pediatrics 133, e1277–e1284. 10.1542/peds.2013-2215 24753535

[B38] Scinicariello F. Buser M. C. (2016). Serum testosterone concentrations and urinary bisphenol A, Benzophenone-3, triclosan, and paraben levels in male and female children and adolescents: NHANES 2011-2012. Environ. Health Perspect. [Epub ahead of print]. 10.1289/EHP150 27383665 PMC5132630

[B39] Seachrist D. D. Bonk K. W. Ho S. M. Prins G. S. Soto A. M. Keri R. A. (2016). A review of the carcinogenic potential of bisphenol A. Reprod. Toxicol. 59, 167–182. 10.1016/j.reprotox.2015.09.006 26493093 PMC4783235

[B40] Smith C. E. Nanci A. (1989). A method for sampling the stages of amelogenesis on mandibular rat incisors using the molars as a reference for dissection. Anat. Rec. 225, 257–266. 10.1002/(SICI)1097-0185 2683870

[B41] Tohmé M. Prud'homme S. M. Boulahtouf A. Samarut E. Brunet F. Bernard L.. (2014). Estrogen-related receptor γ is an *in vivo* receptor of bisphenol A. FASEB J. 28, 3124–3133. 10.1096/fj.13-240465 24744145

[B42] Weerheijm K. L. Jälevik B. Alaluusua S. (2001). Molar-incisor hypomineralisation. Caries Res. 35, 390–391. 10.1159/000047479 11641576

[B43] Weerheijm K. L. Merjare I. (2003). Molar incisor hypomineralisation: a questionnaire inventory on its occurrence in member countries of the European Academy of Paediatric Dentistry (EAPD). Int. J. Paediatr. Dent. 13, 411–416. 10.1046/j.1365-263X.2003.00498.x 14984047

[B44] Woo S. M. Lim H. S. Jeong K. Y. Kim S. M. Kim W. J. Jung J. Y. (2015). Vitamin D promotes odontogenic differentiation of human dental pulp cells via ERK activation. Mol. Cells 38, 604–609. 10.14348/molcells.2015.2318 26062551 PMC4507025

[B45] Ziv-Gal A. Flaws J. A. (2016). Evidence for bisphenol A-induced female infertility: review (2007-2016). Fertil. Steril. 106, 827–856. 10.1016/j.fertnstert.2016.06.027 27417731 PMC5026908

